# Language diversity and challenges to communication in Indian emergency departments

**DOI:** 10.1186/s12245-021-00380-7

**Published:** 2021-09-22

**Authors:** Katherine Douglass, Lalit Narayan, Rebecca Allen, Jay Pandya, Zohray Talib

**Affiliations:** 1 Department of Emergency Medicine, The George Washington University School of Medicine & Health Sciences, 2120 L St., NW Suite 450, Washington, DC 20037 USA; 2 Department of Internal Medicine, The George Washington University School of Medicine & Health Sciences, 2150 Pennslyvania Ave, NW, Suite 8-416, Washington, DC 20037 USA; 3 Present Address: The George Washington University School of Medicine & Health Sciences, 2300 I St, NW, Washington, DC 20052 USA; 4 Present Address: Anne Arundel Medical Center, 2001 Medical Parkway, Annapolis, MD 21401 USA; 5 Present Address: Kings County Hospital Center, 450 Clarkson Avenue, Brooklyn, NY 11203 USA; 6 Present Address: California University of Science and Medicine, 217 E Club Center Dr Suite A, San Bernadino, CA 92408 USA

**Keywords:** Emergency Care, Communication, Language, India

## Abstract

**Background:**

Communication in emergency departments (ED) in India is complicated by the country’s immense language diversity. Prior research has revealed challenges in language and communication as barriers to care. Our objective was to quantify language diversity among clinicians in Indian EDs and better understand issues related to clinician-clinician and clinician-patient communication.

**Methodology:**

A cross-sectional survey of ED clinicians was conducted. Survey participants were recruited in-person and through email at six partner sites in India. ANOVA and binary logistic regression were used for subgroup analysis. Semi-structured interviews were conducted with ED clinicians. Interview data was analyzed using the rapid assessment process to determine predominant themes.

**Results:**

106 clinicians completed the survey. On average, clinicians spoke 3.75 languages. Seventy-one percent used a non-English language to speak to fellow clinicians most of the time, and 53% reported at least one critical incident over the last year where poor communication played a part. Interviews revealed challenges including low health literacy, high patient volume, and workplace hierarchy.

**Conclusions:**

This study is the first to document the impact of language diversity and communication barriers in Indian EDs. The results highlight the need for effective strategies to improve communication between the multiple languages spoken by clinicians and patients.

**Supplementary Information:**

The online version contains supplementary material available at 10.1186/s12245-021-00380-7.

## Background

In the emergency setting, communication is essential to provide efficient and effective patient care, especially given the context of high acuity, limited availability of patient history, and high patient volumes. Prior studies indicate that communication challenges in the Emergency Department (ED) can have a negative impact on quality and safety of care and the patient’s subjective experience [1]. An Australian emergency communication study cited that the main cause of critical incidents in their hospital system, namely adverse events that resulted in patient harm, was poor and inadequate communication between clinicians and patients [2]. Good communication is the foundation of great clinical care in the emergency department. Physician communication is positively correlated with patient adherence to treatment. One meta-analysis indicated that there was a 19% increased risk of non-adherence with patients of physicians who communicated poorly [3]. Additionally, good clinician-patient communication in the emergency department during life-threatening cardiac events has been associated with decreased subsequent post-traumatic stress reactions [4].

In India, linguistic alignment of providers and patients is even more complex due to the immense language diversity that exists within the country. There are over 22 official languages that are spread over the regions of India, and at least 122 different spoken languages [5]. While many of these languages are regional, migration patterns continue to contribute to a diversity of language in any local setting. Similar to other countries, a physician in training in India may grow up in one region, obtain their medical degree in another, and complete their specialty training in yet again another region. Unique to India, however, each of these regions is likely to have a different primary spoken language. Therefore, physicians in training in India are not only learning medicine, they are often learning the languages of their patients along the way. These factors result in a multilingual health care environment where ensuring language alignment presents a challenge.

Language barriers in the health care setting are neither a novel nor a foreign problem. In the United States individuals with limited English proficiency are documented to have worse healthcare access and report lower quality of care when compared to individuals proficient in English [6]. Additionally, language barriers have been reported as one of the greatest causes of health care disparities in a cross-sectional study of pediatric emergency departments [7]. Effects of language barriers can range from misunderstandings to compromising quality of care [8]. An in-depth language and culture study performed at a pediatric hospital in South Africa investigated the communication between English-speaking doctors and Xhosa-speaking patients and parents. The study documented that even when physicians and parents were using the same words, those words held different meanings for each group. This led to what they concluded to be a clinically significant discordance in understanding. Thus begins to unravel the cultural complexities that are entwined with language diversity [9].

Communication in the health care setting is not only stymied by language diversity, but a host of barriers that include health literacy. An extensive 2011 systematic review of the literature reported the low health literacy is associated with poorer health outcomes and poorer use of healthcare services [10]. A recent study sampling English-speaking and Spanish-speaking ED patients to investigate health literacy using language congruent tools found that 93% of Spanish-speaking patients sampled had limited health literacy [11]. Self-reported reading ability and years of school completed have been shown to be adequate predictors of health literacy [12].

This study was undertaken to better understand the challenges to effective communication in the ED in India, including but not limited to language. The field of Emergency Medicine is in its infancy in India. Emergency Medicine was recognized as an independent specialty by the Indian government in 2009. Pre-hospital care and trauma responses have been described as “disorganized and inadequate” by India’s own emergency experts [13]. Government-sponsored EM training programs only produce 48 emergency physicians each year to serve a population of over 1.3 billion. To contribute to closing this gap in education and training, some private hospitals in India have partnered with US academic institutions, including the Ronald Reagan Institute of Emergency Medicine at the George Washington University, to provide post-graduate emergency medicine training for physicians. Our department has affiliations at numerous hospitals across India [14]. A previous study and first-hand experience have revealed significant gaps in language and communication in Indian EDs. This mixed-methods study aims to examine communication issues experienced by health care providers at six hospitals in India.

## Methods

A cross-sectional survey and semi-structured interviews of ED clinicians was conducted from May to July 2017. Study sites were recruited from an open invitation distributed to program directors at the ten education and training partnership programs active at the time of the study. Six sites were chosen based on positive responses from the program director combined with convenience for the research team, including Aster CMI in Bangaluru, BGS Global in Bengaluru, Aster in Wayanad, Aster MIMS in Kozhikode, Baby Memorial in Kozhikode, and Aster Kottakkal. Survey and interview participants were recruited via convenience sampling of physicians, nurses, and paramedics working in the ED. ANOVA and binary logistic regression were used to perform subgroup analysis. The study design and materials were submitted to the Institutional Review Board of our institution and determined to be exempt from review.

The interview guide was adapted from previous research examining the impact of language diversity in a trilingual E D[15]. Student researchers were trained by the study lead in interview procedures. The interview guide was piloted and minor changes were made based on feedback for clarity and language. See Additional file 1: Appendix A for the interview guide. Verbal consent was obtained from all interviewees. Interviews were conducted in person and recorded by the student interviewer using a voice recording device. Most transcriptions were completed by the interviewer, and a transcription service was used to transcribe the rest (www.datagainservices.com). All personal identifying information was withheld from transcriptions, and transcriptions were securely stored and only accessed by study team members.

Transcriptions were analyzed using a rapid analysis technique to identify predominant themes by the non-student members of the research team [16]. Two co-investigators developed a matrix in Microsoft Excel identifying coded domains related to each interview question. Each interview was deductively analyzed using the established codes. To assess consistency across the analysis team, each of the members performed rapid summary analysis of two common transcripts. The remaining transcripts were divided among study team members for analysis. The final matrix compiled all results and provided a visual summary of major themes and subthemes across stakeholder groups.

## Results

### Quantitative data

One hundred six clinicians completed the survey including 42 doctors (9 consultants and 33 post-graduate trainees), 45 nurses, and 19 paramedics. See Table 1 for survey results. On average, respondents spoke 3.75 languages. None of the respondents were monolingual. Fluency in the majority language at the hospital was reported by 93% of doctors, 84% of nurses and 95% of paramedics. Fluency in English was reported by 100% of doctors, 71% of nurses and 63% of paramedics. Type of clinician, age, gender, and time in clinical practice did not predict the number of languages spoken or fluency in the majority language. Doctors were more likely to report fluency in English, compared to other clinicians (*p* < 0. 003).

**Table 1:** Survey results

		Overall (***n***=106)	Consultants (***n***=9)	Residents (***n***=33)	Nurses (***n***=45)	Paramedics (***n***=19)
Gender	Male	83	9	27	28	19
	Female	23	0	6	17	0
Age	< 20	3	0	0	1	2
	20–30	76	1	19	39	17
	31–40	23	5	13	5	0
	> 40	3	3	0	0	0
Time in clinical practice	< 4 years	63	2	13	32	16
	5–9 years	37	3	20	11	3
	> 9 years	6	4	0	2	0
Percent of clinical practice time in EDs	< 25%	6	0	2	3	1
	26–50%	34	2	6	18	8
	51–75%	40	4	16	13	7
	> 76%	26	3	9	11	3
Training in communication?	Yes	74	7	18	33	16
	No	27	2	13	10	2
Current work hours per week	< 40	9	2		6	1
	40–80	87	7	29	37	14
	> 80	6	0	2	1	3
Do you explain diagnoses to the patient or family?	No	18	0		15	3
	Yes	84	9	31	29	15
How many languages do you speak?	1	0	0	0	0	0
	2	12	1	1	7	3
	3	35	2	11	15	7
	4	32	3	12	13	4
	5	22	3	6	9	4
	6	5	0	3	1	1
Are you fluent in English?	No	19	0	0	12	7
	Yes	86	9	33	32	12
If yes, in which of the following ways are you fluent?	Yes spoken	71	8	33	19	11
	Yes reading	75	9	31	17	8
	Yes writing	67	7	29	27	6
Are you fluent in the language spoken by the majority of patients?	No	10	0	3	6	1
	Yes	95	9	30	38	18
If yes, in which of the following ways are you fluent?	Yes spoken	86	7	30	32	17
	Yes reading	64	4	24	25	12
	Yes writing	59	4	21	22	12
What language do you speak most of the time with your fellow providers in the Emergency Department?	English	28	6	13	9	
	Malayalam	61	2	9	32	18
	Hindi	3		3		
	Kannada	5	1	1	3	
	Tamil	2		2		
	Other	4		3		1
During which of the following instances do you think communication can be a problem?	Triage	38	2	9	17	10
	Patient interview	43	5	14	16	8
	Handoff	18	8	8	2	
	ED physician-consultant interaction	17	2	11	4	
	Physician-nurse interaction	15	2	8	4	1
	Supervisor-trainee interaction	6	2	3		1
	Physician-paramedic interaction	4	2	2		
Are you aware of any critical incidents in the past 12 months in the Emergency Department where you work in which poor communication played a part?	No	46	3	6	26	11
	Yes 1	26	3	12	8	3
	Yes 2–5	26	3	11	9	3
	Yes > 5	4	0	2	1	1
Which of the following barriers may have played a role in that poor communication?	Language	30	4	10	14	2
	Noise	15	0	6	8	1
	Pt volume	25	2	13	4	6
	Time constraints	32	5	11	12	4
	Role identification	13	3	5	4	1
	Long working hours	23	1	12	9	1
	Difference in knowledge	28	5	15	6	2
Do these long working hours affect your ability to communicate effectively?	Always	23	1	8	1	2
	Sometimes	56	5	18	25	8
	Rarely	9	1	4	3	1
	Never	14	2	1	1	7
When you explain English medical knowledge to patients in a different spoken language, do you think that information is lost or changed?	Always	7	1	1	2	3
	Sometimes	61	6	22	24	9
	Rarely	22	1	6	12	3
	Never	12	1	2	6	3

Seventy-one percent of respondents reported that they used a non-English language to speak to their fellow clinicians most of the time. Sixty-four percent felt that information was lost or changed when English medical knowledge was explained in a different language. Seventy-three percent reported prior training in communication, including a majority of paramedics and nurses. Fifty-three percent reported at least one critical incident over the last year where poor communication played a part. Time constraints, language, and differences in medical knowledge were the most frequently identified barriers in these incidents. Seventy-seven percent of respondents reported that long working hours either always or sometimes affected the ability to effectively communicate. Sixty-six percent of respondents reported that information is always or sometimes lost in translation when explaining medical concepts to patients in another language.

### Qualitative data

In total 106 interviews were completed and analyzed. See Table 2 for descriptions of respondents. Thematic analysis of the results revealed two major sub-types of communication in both the communication between patient and provider, and communication between providers. Within each type of interaction, we found themes of language discordance and concordance. Elaborating on this, when the two parties communicating are speaking different languages, there were challenges. However, even when the two parties communicating were speaking the same language, significant challenges in communication were still identified.

**Table 2:** Interview participants

	Consultants (14)	Residents (43)	Nurses (19)	Paramedics (17)
Aster CMI, Bengaluru (12)	4	4	4	0
BGS Global, Bengaluru (9)	2	5	2	0
Aster, Wayanad (17)	3	8	4	2
Aster MIMS, Kozhikode (27)	3	14	6	4
Baby Memorial, Kozhikode (24)	2	10	2	10
Aster, Kottakkal (4)	0	2	1	1

#### Patient-provider communications

Various issues were identified in interactions between patients and providers, with illustrative quotes provided in Table 3. In communication scenarios between patients and providers, there are obvious challenges in cases of language discordance. We identified 20 clinicians in our interviews who reported not being fluent in the majority local language. Questioning revealed that these clinicians were, for the most part, from a different state and were training or working in a hospital in which the language spoken by the majority was different or unknown to them. Additional challenges were described when patients spoke a different language than the local language, and the absence of wide-spread translator services was noted. Even in locations where translator phones were available, these services were not available at all times.

**Table 3:** Patient-provider communication

Consultant “So a fluent, well taken history is very important in coming to a conclusion or coming to a diagnosis. So if we are not fluent in the languages of the patient it’s going to affect the diagnosis.”	
Post-graduate trainee “Now here in Kerala many people are coming from Bengal, Assam, those kinds of areas. So they don’t speak Hindi also. They speak Assamese, Bengali. It’s very hard to pick up. In those situations. Those situations are hard, we just - just rule out some emergency situation…”	
Post-graduate trainee “During my initial stages of residency I wasn't able to understand any of the complaints, what the patient is saying. I was dependent on nursing staff. Sometimes even I can’t tell the difference between diarrhea and constipation”	
Post-graduate trainee “Not exactly, because in MIMS we get international patients mostly, the Arabic patients. So there will be communication issues. So we need a translator. So in odd hours we won't be having a translator, that time maybe.”	
EMS/Tech “There are a lot of rural people here. They don’t understand. Their education is not enough. They’re illiterates”	
EMS/Tech “It’s very difficult to communicate with them. Sometimes they are asking some doubts and it's very difficult to communicate with them, because we have a medical term, but it's difficult to translate to our mother tongue.”	
EMS/Tech “In triage there are lot of problems coming, because people are not aware about triage and they are always irritated. They are coming with excessive pain, but they are not allowing us to triage. There is lot of communication problem.”	

Beyond the issue of not speaking the same language, the issue of inadequate communication at various times in the medical experience was also a common barrier. For example, challenges occur when there was a mismatch between patient expectations and actual processes, such as during triage when a patient expectation of immediate evaluation may clash with triage protocols. Additionally, health literacy was a factor in patients’ understanding of a medical situation. Explaining a complex medical process to a person with less experience or education is challenging. This was sometimes even compounded by some languages not having analogous words to explain medical phenomenon. Providers would often have to rely on creating metaphors to explain physiologic processes by evoking shared understanding of nature, flowers, or trees.

Another commonly described theme was difficulty with what are known as “bystanders.” Bystanders are the family and friends of a patient in the emergency department. It was noted that communication could often times be complicated by the presence of many people with different opinions, expectations, and agendas.

#### Provider-provider communications

Various issues were also identified in interactions between healthcare providers, with illustrative quotes provided in Table 4. In the context of language discordance, resident physicians were most likely to not be fluent in the local language. This was most commonly an issue between resident physicians and nurses or paramedics, as the nurses and paramedics are generally fluent in the local language whereas resident physicians may not be fluent in the local language, and sometimes are more comfortable speaking in English.

**Table 4:** Provider-provider communication

EMS “...the doctors or staff who don’t speak Malayalam language, it's very difficult to communicate with them.”	
Resident “My colleagues do understand my language completely, but the nursing staffs sometimes don't understand me. They have to work for me, like two weeks or something, then they get to understand what I am saying”	
Nurse “Sometimes lack of confidence to speak into, because if we don’t know English, we may hesitate to speak in English to the person who knows English”	

In communication between providers when the language itself was not an issue, some respondents describe particular times during which communication was a more prominent issue in patient safety, such as busier times of day or during patient handover. Additionally, there are some challenges described in the integration of the electronic medical record and verbal communication, such as integration of verbal and written orders. Some respondents also reported issues with hierarchy, both in the ranks of physicians as well as between physicians and other staff members impacting comfort level in communicating different ideas or questions regarding clinical care. However, many respondents also reported camaraderie among the providers with a family mentality and team approach to patient care, suggesting that better relationships among providers facilitate more effective communication during difficult or stressful patient encounters or scenarios.

## Discussion

This study serves as an introduction to the vast complexities of communication that exists in Indian Emergency Departments. It is critical to note, that despite the language diversity and obstacles described, these institutions still provide a high quality of care. The challenges that the language diversity and the nature of working in an ED bring to communication are certainly barriers, but they are not insurmountable.

Our study is the first to document language diversity in Indian EDs. Important findings include the common use of non-English language in clinician to clinician communication and the frequent perceived loss of information in clinician-patient communication. The reported rates of critical incidents linked to poor communication are higher than reported in comparable studies and warrant further research and action. Additionally, the interviews revealed the challenges that bystanders can bring and the gap that exists in health literacy in the general population.

## Limitations

There are several limitations in our study. The first was the site selection. The study was only done at private partner institutions and with individuals that agreed to participate. The hospitals also did not include any public hospital sites. This presents a risk of sampling bias as well as convenience bias. The sites that we conducted the study at were also limited in number and concentrated in the southern and western parts of India. Specifically, four out of the six sites were in the state of Kerala which has the highest literacy rates in the country. Regional variability in site selection may have provided a more diverse physician profile and perhaps different challenges to communication in areas with lower literacy levels. The original study design did include surveying hospitals in northern parts of India, but the data collection had to be cut short on account of the surveyor contracting dengue fever. Lastly, the data collected only considers the provider’s point-of-view. Including patients in further studies could help highlight and clarify the challenges brought up through the provider interviews.

## Conclusions

Language and communication play an integral role in healthcare delivery in the ED. This is the first study to analyze communication issues in the EDs in India; the results highlight that communication is affected by language when there is a mismatch in language between patients and providers. That said, even when language discordance is not a problem, there are still challenges that compromise effective communication. The findings of our study suggest that interventions aimed at improving communication in Indian EDs will have to account for the diverse, multilingual nature of Indian medical practice and the difficulties bridging the gap of health literacy between clinicians and patients. After receiving training in English, Indian clinicians are challenged to speak multiple languages as they communicate with patients, bystanders, and fellow clinicians each day. Given the scale of the Indian health system, possible solutions cannot rely solely on the use of professional interpreters. as in the US. Moving forward, incorporating language training as part of health professional education, and greater health information interventions for the public would be worth pursuing.

## Supplementary Information

**Additional file 1:.** Appendix A.

## Data Availability

Not applicable
