# Supplementary material for: Language diversity and challenges to communication in Indian emergency departments
Source: Int J Emerg Med. 2021 Sep 22;14:57. doi: 10.1186/s12245-021-00380-7 (PMC8459521; doi:10.1186/s12245-021-00380-7)
Supplement: Supplementary file 1 — Additional file 1:. Appendix A. [file 12245_2021_380_MOESM1_ESM.docx]

Interview Guide – Communication Project – 4/26/17

Semi-Structured Interview Guide:

(introduce yourself, provide the consent form, etc)

Demographics:

1. Describe yourself:
   1. How many languages do you speak?
2. Describe your current job:
   1. What is your job title? How long have you been in your current position?
3. Describe your current institution:
   1. For how long have you be working at your current institution?
   2. Is the local language (majority spoken language in your hospital) a new language for you, or one that you speak fluently?

Communication between patients and providers:

1. Can you provide an example of a scenario when there was a communication issue with a patient that affected clinical care? If yes, please describe.
   1. Can you describe what led to the communication issue?
2. When you think generally about communication in the ED, is language a factor? Tell me more…
3. What about barriers other than language? (Prompts could include time pressures or patient volumes, chaos and noise in the ED, health literacy, training on communication etc). If yes please describe; tell me more
4. Are there specific times during a patients’ experience during which communication issues are more likely to occur? (i.e. triage, critical situations?). If yes, please describe

Communication between providers:

1. Can you provide an example of a scenario when there was a communication issue between providers?
   1. Can you describe what led to the communication issue?
2. Was language a factor? Tell me more..
3. What about barriers other than language? (prompts could include time pressures or patient volumes, provider role perception, cultural/interpersonal issues, etc). If yes please describe
4. Are there specific times during a patients’ experience during which communication issues between providers are more likely to occur? (i.e. handoffs, triage, critical situations?). If yes, please describe
5. Are there examples of communication between types of providers which are more prone to communication issues? (i.e. nurse to resident, residents to attending, consultant to nurse)?
